# Pipkin Type I and II femoral head fractures: internal fixation or excision?—from the hip arthroscopy perspective

**DOI:** 10.1093/jhps/hnad002

**Published:** 2023-02-23

**Authors:** Chung-Yang Chen, Shan-Ling Hsu, Chi-Hsiang Hsu, Hao-Chen Liu, Yu-Der Lu

**Affiliations:** Department of Orthopaedic Surgery, Kaohsiung Chang Gung Memorial Hospital and Chang Gung University College of Medicine, No 123, Dapi Rd., Niaosong Dist., Kaohsiung 83301, Taiwan; Department of Orthopaedic Surgery, Kaohsiung Chang Gung Memorial Hospital and Chang Gung University College of Medicine, No 123, Dapi Rd., Niaosong Dist., Kaohsiung 83301, Taiwan; Department of Orthopaedic Surgery, Kaohsiung Chang Gung Memorial Hospital and Chang Gung University College of Medicine, No 123, Dapi Rd., Niaosong Dist., Kaohsiung 83301, Taiwan; Department of Orthopaedic Surgery, Kaohsiung Chang Gung Memorial Hospital and Chang Gung University College of Medicine, No 123, Dapi Rd., Niaosong Dist., Kaohsiung 83301, Taiwan; Department of Orthopaedic Surgery, Kaohsiung Chang Gung Memorial Hospital and Chang Gung University College of Medicine, No 123, Dapi Rd., Niaosong Dist., Kaohsiung 83301, Taiwan

## Abstract

The treatment of patients with femoral head fractures with regard to fixation versus excision is controversial. This study aimed to compare the results of fixation and excision in hip arthroscopy–assisted surgery. This retrospective study included adult patients with femoral head fractures who were treated with hip arthroscopy surgery from March 2016 to April 2020, with a minimum follow-up of 24 months. The patients were divided into two groups: Group 1 (fixation group) and Group 2 (excision group). To compare the therapeutic effects between the two groups, clinical and radiographic outcomes, operative time, pain score, length of hospital stay after surgery and related complications were investigated. There were 13 (mean duration, 47.5 months; range, 24–72 months) and 8 (mean duration, 48.6 months; range, 26–74 months) patients in the fixation and excision groups, respectively. The excision group had better functional results than the fixation group in terms of the median modified Harris hip score (*P* = 0.009). No significant differences were observed in operative time, pain score or hospital stay after surgery between the two groups. Further, no osteonecrosis of the femoral head or traumatic arthritis occurred in either group. A piece of fracture fragment >2 cm can be considered for hip arthroscopy–assisted internal fixation, whereas the others can be removed. The excision group had better outcomes than the fixation group. Hence, hip arthroscopy–assisted internal fixation or excision of bony fragments led to satisfactory short-term clinical and radiological results for the treatment of Pipkin Type I and II femoral head fractures.

## INTRODUCTION

Femoral head fractures are complex lesions that may have severe consequences. They are often the result of high-energy trauma and are associated with significant soft tissue and intra-articular injuries. Several classification systems for femoral head fractures have been described previously. The most widely used system is the Pipkin classification [1], which is based on the location of the head fragment related to the fovea and associated lesions on the femoral neck or acetabulum. The proposed treatment methods for Pipkin Type I or II bone fragments are excision and fixation [6]. However, associated flaws with these methods hinder accurate decision-making.

Several studies addressed important issues regarding the surgical outcomes of Pipkin Type I and II femoral head fractures [2–4]. However, there are controversies regarding the indications for fixation or excision of the fracture fragment and the appropriate surgical approach [3, 5, 6]. Open reduction seems to result in extensive joint exposure and represents a greater risk of affecting femoral head blood supply. Complications associated with surgery for femoral head fractures, such as heterotopic ossification (HO), osteonecrosis of the femoral head (ONFH) and arthritis, have been reported [2, 7–12]. Different therapeutic options can be employed in the treatment of these lesions.

Arthroscopy-assisted surgery in patients with Pipkin Type I or II femoral head fractures has recently been reported. With the development of arthroscopic surgery, minimally invasive percutaneous surgery offers several advantages over other surgical techniques [13–16]. A comparison of the outcomes of internal fixation and excision of femoral head fracture-dislocations after hip arthroscopy–assisted surgery is lacking. In this study, we retrospectively collected and analysed data from patients with Pipkin Type I or II femoral head fractures who underwent hip arthroscopy–assisted surgery. We hypothesized that arthroscopy-assisted surgery is equivalent to open techniques, whether fixation or excision, and should have no effect on the results of femoral head fractures in the short-term follow-up.

## MATERIALS AND METHODS

Institutional review board approval was obtained using the umbrella protocol for retrospective cohort studies of the Chang Gung Medical Foundation (IRB number: 201700816B0). Patients with femoral head fractures were treated with hip arthroscopy between March 2016 and April 2020. The inclusion criteria were displaced Pipkin Type I, II or other types of femoral head fractures that were successfully treated with hip arthroscopy. The procedure was thoroughly explained to all patients, who provided informed consent and then underwent hip scope–assisted surgery. The exclusion criteria were Pipkin Type III or IV fractures and fractures managed by open surgery or other non-operative means. A single surgeon performed all surgeries. Another surgeon collected the subjects’ data and analysed the clinical and radiographic data.

The patients were divided into the following two groups according to surgical approach: (i) Group 1, underwent (fixation group), and (ii) Group 2 (excision group). The indications for surgical internal fixation for femoral head fracture-dislocation were a large fracture fragment and displacement >2 mm or Pipkin Type II on a computed tomography (CT) scan. The indications for excision were a fracture fragment <2 cm or a comminuted and irreducible fracture fragment. The modified Harris hip score (mHHS) and Thompson and Epstein outcome criteria (T–E criteria) [17] were used to evaluate the functional and radiologic outcomes. CT scans with three-dimensional (3D) reconstruction were performed to assess the condition of the fracture site before and 3 months after surgery in the fixation group. After the index surgery, plain pelvic anteroposterior and lateral hip view scans were obtained. The Ficat classification of ONFH [18], the Brooker classification of HO [19] and the joint space width of traumatic arthritis <2 mm were used to assess the radiological outcome. Patient age, sex, operative time, the first 24-h pain score (visual analogue scale [VAS]) and hospital stay after surgery were recorded in a custom-made database.

### Surgical methods

**Fig. 1.**
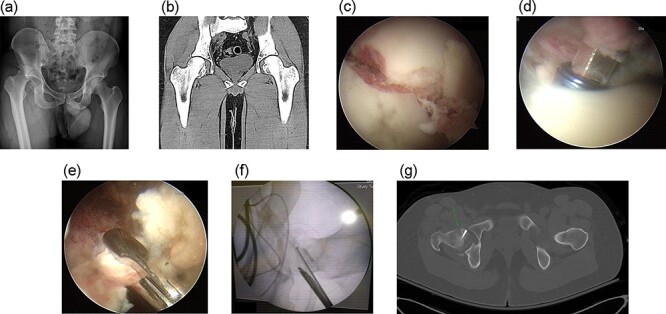
A 28-year-old man (Case 4) with a Pipkin Type I fracture of the right hip with posterior dislocation of the femoral head. (a) The anteroposterior X-ray image of the injury in the right hip. (b) The pre-operative anteroposterior CT scan of the right hip after closed reduction showing inferior mini displacement of the femoral head fragment. (c) Fluoroscopy showing fracture reduction of the femoral head after guidewires insertion and (d) after fixation of screws during the surgery. (e) Concomitant intra-articular hip lesions secondary to traumatic hip dislocation can also be treated. (f) Fluoroscopic examinations confirmed the successful and secure fixation of the fracture fragments without protrusion. (g) The post-operative CT scan showing acceptable internal fixation and congruity of the femoral head ).

**Fig. 2.**
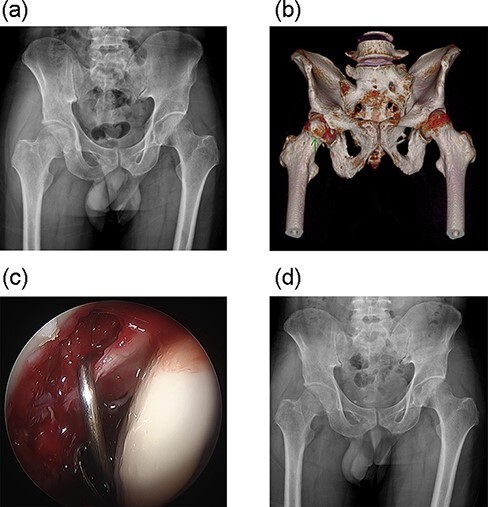
A 40-year-old man (Case 17) was transferred from other hospital due to persisted hip pain even after 3 months’ conservative treatment. (a) The radiology of the right hip showed incongruent joint with bony fragments. (b) 3D-CT showed that the fragments of Pipkin Type I femoral head fracture were small and comminuted and were excised. (c) The intercalated bone fragment that made joint incongruent was removed by arthroscopy. (d) The radiology showed symmetric hip joint space after the removal of bony fragment.

Under general anaesthesia, each patient was placed in the supine position on a fracture table with intermittent traction, which limited the traction time to <2 h. The perineal post was well padded and folded eccentrically to minimize the risk of pudendal nerve compression. We depicted the anatomic locations of bony prominences to identify portal locations and guidewire insertion sites: the anterior superior iliac spine and the greater trochanter. The position of the femoral artery was marked on the skin using ultrasonography. We used three standard portals to visualize the hip joint using a 70° arthroscope. A surgical grasper, elevator and probe were used in the joystick method to reduce fracture fragmentation when the fracture site was in the superior area in the hip external rotation position in Group 1. After thorough reduction of the fracture site, percutaneous guidewires were inserted into the femoral head via blunt dissection of the muscle under fluoroscopic guidance. The insertion sites were located between the anterior portal and the anterior superior iliac spine. The tips of the wires were guided to the fracture site with the aid of fluoroscopy and arthroscopy. The tip was as close as possible to the centre of the fracture fragment. The large fracture fragment (>2 cm on CT scan) or Pipkin Type II fracture was fixed with two or three 2-mm Herbert screws engaged in the subchondral bone below the articular surface. Dynamic fluoroscopy determined the screws’ length without protrusion after fracture fragment fixation (Fig. 1). The stability of the fracture site after reduction was confirmed by probes and video footage acquired using a scope through different portals. If the bony fragment was <2 cm or comminuted and irreducible, excision was performed. Small osteochondral fragments in the hip joint and labral tears were too comminuted to be repaired and excised during the surgery (Fig. 2), following which the wound was closed and no suction drains were placed in the joint.

First-generation cephalosporins were administered as antibiotic prophylaxis prior to anaesthesia and 24 h after surgery. The patients were able to walk with toe-touch weight-bearing using crutches the day after surgery, and the patients were discharged when the wound condition was stable. The patients were assessed clinically at 1, 3, 6 and 12 months after discharge. Thereafter, follow-up was conducted annually.

### Statistical analyses

The categorical and continuous variables of the two groups were compared using Fisher’s exact test and Mann–Whitney test, respectively. All *P*-values were two-sided. The level of statistical significance was set at *P*  < 0.05. Statistical analyses were performed using the Statistical Package for the Social Sciences (SPSS 20.0; IBM, New York, USA).

## RESULTS

Twenty-six patients with femoral head fractures who underwent hip arthroscopy were included in this study. Among these, five were excluded: two cases (Case 6 and 22) due to failure of hip arthroscopy surgery and requirement of open surgery, while the other cases had Pipkin Type IV fractures with combined open and hip arthroscopy surgery (Table I). Finally, 21 patients were enrolled in this study, of whom 13 patients (11 were Pipkin Type I and 2 were Pipkin Type II) in Group 1 underwent fixation and 8 (7 were Pipkin Type I and 1 was Chiron’s Type V) in Group 2 underwent excision. The fractures were located in the infra-foveal area, with the exception of Case 21, which was a superior femoral head chip collapse fracture (Chiron’s Type V) [20]. The patients were followed up at the clinic for a mean duration of 47.5 months (range, 24–72 months) in Group 1 and 48.6 months (range, 26–74 months) in Group 2. Plain radiographs and 3D-CT images obtained after closed reduction in the emergency room showed displaced femoral head fractures. In addition, one (Case 5) patient in Group 1 and one (Case 21) patient in Group 2 had an ipsilateral femoral shaft fracture and underwent staged open reduction and internal fixation surgery. Another patient (Case 18) in Group 2 had an ipsilateral tibial eminence fracture and was simultaneously treated with knee arthroscopy–assisted internal fixation.

**Table I.** Patient characteristics

*P. No.*	*Age*	*Sex*	*Pipkin type*	*Chiron’s t*	*Associated injury*	*OP method*	*OP time (mins)*	*Hospital stay (days)*	*VAS*	
Fixation group (Group 1)										
1	19	M	I	IIB	Nil	Arthroscopic fixation	230	3	5	95.7
2	24	M	II	IIIA	Nil	Arthroscopic fixation	320	3	3	95.7
3	48	M	I	IIIB	Nil	Arthroscopic fixation	230	4	3	95.7
4	28	M	I	IIB	Nil	Arthroscopic fixation	210	4	5	93.5
5	20	M	I	IIIB	Femoral shaft fr.	Arthroscopic fixation		4	5	92.4
6	17	F	I	IIB	Nil	Open fixation				
7	26	M	I	IIIB	Nil	Arthroscopic fixation	220	2	4	93.5
8	19	M	I	IIB	Nil	Arthroscopic fixation	200	4	3	93.5
9	21	M	I	IIA	Nil	Arthroscopic fixation	195	3	2	92.4
10	32	M	I	IIB	Nil	Arthroscopic fixation	190	3	3	88
11	45	F	II	IIIB	Nil	Arthroscopic fixation	325	4	3	93.5
12	19	M	I	IIA	Nil	Arthroscopic fixation	250	4	3	92.4
13	22	F	I	IIB	Nil	Arthroscopic fixation	225	15	5	93.5
14	41	M	I	IIA	Nil	Arthroscopic fixation	150	17	3	93.5
Excision group (Group 2)										
15	41	M	I	IIA	Nil	Arthroscopic excision	120	4	3	95.7
16	39	F	I	IIA	Nil	Arthroscopic excision	230	3	3	95.7
17	40	M	I	IIA	Nil	Arthroscopic excision	225	2	3	95.7
18	32	M	I	IIA	Tibial eminence fr.	Arthroscopic excision		3	3	95.7
19	19	M	I	IIB	Nil	Arthroscopic excision	195	3	3	95.7
20	19	M	I	IIB	Nil	Arthroscopic excision	200	3	5	95.7
21	18	F	other	V	Femoral shaft fr.	Arthroscopic excision				
22	18	F	I	IA	Nil	Open excision				
23	22	F	I	IIA	Nil	Arthroscopic excision	265	3	3	93.5

OP: operation, fr.: fracture.

The excision group had better functional results than the fixation group in terms of the median mHHS (*P* = 0.009). The results are presented in Table II. In case of Pipkin Type I and II fractures, the median mHHS was 93.5 (range, 88–95.7) in Group 1 and 95.7 (range, 93.5–95.7) in Group 2. Based on the T–E criteria, the overall results for the 13 cases in Group 1 were excellent in 11 (85%) and good in 2 (15%) cases. In Group 2, all results were excellent (100%) at the latest follow-up visit. No significant differences were observed in age, sex, type of fracture, operative time, VAS score or hospital stay after surgery between the two groups.

**Table II.** Statistical analyses of patients characteristic, outcome and radiological results who had femoral head fracture-dislocations treated with hip scope–assisted surgery

	*Median (Q1–Q3) or number (%)^a^*		
*Variables*	*Fixation group (n = 13)*	*Excision group* *(n = 8)*	*P value*
Age at the time of operation (years)	24 (20–32)	27 (19–39.25)	0.798
Sex			0.259
Male	11 (85)	5(62.5)	
Female	2(15)	3(37.5)	
Pipkin type			0.713
I	11 (85)	6 (87.5)	
II	2 (15)	0 (0)	
Other	0 (0)	1 (12.5)	
OP time (min)^b^	222.5 (198.75–235)	212.5 (196.25–228.75)	0.605
Hospital stay after surgery^c^ (days)	4 (3–4)	3 (3–3)	0.133
VAS score	3 (3–5)	3 (3–3)	0.481
mHHS^d^	93.5 (92.4–93.5)	95.7 (95.7–95.7)	0.009
T–E score^d^	Excellent, 11 (85%) Good, 2 (15%)	Excellent, 7 (100%)	0.521

a The continuous and categorical variables were expressed as medians (Q1–Q3) and counts (percentages), respectively.

b Case 6, 12 and 17 were excluded for simultaneous ipsilateral femoral shaft or tibial eminence fracture fixation, respectively.

c Case 22 and 23 were excluded for prolonged care about additional injury during admission.

d Case 17 was excluded for Chiron’s Type V femoral head fracture.

Blood loss was minimal in all patients, except for losses of ∼150 cc in Case 5, 18 and 21. No wound infection or severe pain (VAS score >7) was observed after the surgery. Only one patient (Case 15) had Grade I HO. None of the patients exhibited early osteoarthritis or ONFH. Fracture union was achieved in Group 1 patients without any additional procedures within 3 months according to follow-up radiographs. Post-operative CT scans with 3D reconstruction verified femoral head congruency with no screw migration. All patients tolerated the procedure well without complications related to hip arthroscopy surgery, with the exception of Case 5, who had a femoral head and shaft fracture and suffered temporal sciatic nerve palsy, who had recovered at the 3-month follow-up.

## DISCUSSION

**Fig. 3.**
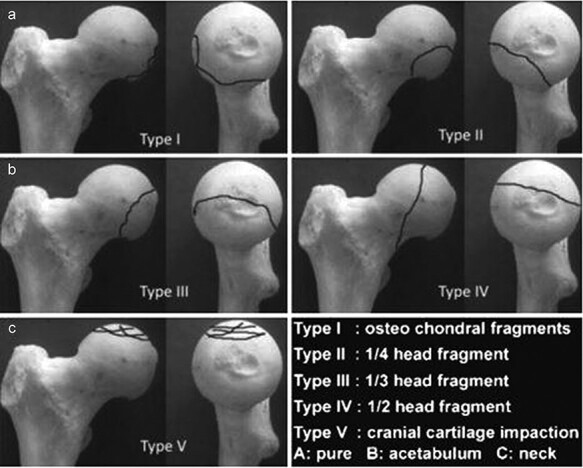
Chiron classification (2004). The fracture types are described based on the size of the fragments and subsequently subdivided into three groups, depending on the location of the fragment and associated acetabular or femoral neck fracture. *Source*. Image with permission of Elsevier [20].

Most studies concur that a large fracture fragment or fragment cephalad to the fovea of the femoral head should be fixed rigidly [3, 5, 21, 22]. The controversy lies in the bony fragments caudal to the fovea. Many classification systems for femoral head fractures have been developed; however, these systems are inadequate [17, 20, 23–25]. The most important factors that consider the size of the fragments and any associated injuries were proposed. The Chiron classification system [26] describes the various fragments better (Fig. 3). But there is still no distinct reference to determine surgical fixation or excision based on the size of the fracture fragment [26]. In the present work, our decision regarding fixation or excision was mainly dependent on the measurement of the fragment as assessed on a pre-operative CT scan. In Pipkin Type I, in which the fragment was >2 cm, hip scope–assisted reduction and internal fixation were considered. On the other hand, if the fragments were small or comminuted, they were excised. In cases where hip scope–assisted surgery did not achieve good anatomic reduction, open excision was performed.

There is still no consensus regarding the optimal conservative treatment or surgery for femoral head fractures. Previous literature suggests that conservative methods should be considered initially, and surgery is indicated when reduction of the fracture or stable joint is not achievable by closed measures [22, 27]. Closed reduction and skin or skeletal traction are treatment options for some femoral head fracture patients with anatomic reduction of hip dislocation, absence of intra-articular loose osteochondral fragments and joint stability [1, 5]. The patients must tolerate an extended period of traction [26]. Henle et al. showed that poor anatomical reduction of femoral head fracture-dislocation after closed reduction and malunion can lead to osteoarthritis and ONFH [28]. The poor results after conservative treatment have been reported [29, 30]. Recently, more studies have revealed that treatment after femoral head fractures has changed over the past few decades. Early surgical intervention for Pipkin Type I fractures can result in favourable outcomes [6, 31]. In our study, early hip scope–assisted surgical intervention shortened the duration, for which patients were bedridden and achieved good short-term clinical results in displaced femoral head fractures. Although good results mentioned in the literature cannot be ignored regarding cases where anatomic reduction is possible via closed means [32–34], hip scope–assisted surgery may be an accessible intervention if surgical treatment is required to treat such injuries.

If surgery is selected, the surgical approach needs to be decided. A systematic review and meta-analysis of Pipkin Type I fracture suggested that surgical excision has the best functional outcomes and fixation may have a higher avascular necrosis rate [35]. Analysis of our outcomes after the application of hip scope–assisted surgery for the treatment of Pipkin Type I and II femoral head fractures demonstrated excellent and good results after either fragment excision or fixation. There were no significant differences in operative time, VAS score or hospital stay after surgery between the two groups. The excision group showed better outcomes than the fixation group according to mHHS (*P* = 0.009), which is similar to open surgery [2]. We also had two patients with good clinical results in the fixation group owing to mild limited hip flexion; otherwise, all had excellent results in both the groups based on the T–E criteria. Even biomechanical cadaveric studies have revealed no adverse long-term clinical implications after excision of a small part (one-third) of the non–weight-bearing surface of the femoral head, such as Pipkin Type I [36]. Many factors, including the severity of fracture type, the small number of cases and the lack of long-term outcome evidence, are contradictory to making conclusions regarding the success of this treatment option. However, based on the present results, managing Pipkin Type I and II femoral head fractures requiring surgery with hip arthroscopy–assisted fixation or excision is a safe and minimally invasive option to achieve good short-term clinical outcomes.

This study had several limitations. First, only a small number of patients were enrolled, and the follow-up period was shorter. Second, the study consisted of a single centre and one surgeon; thus, gradual experience reflected the outcome.

## CONCLUSION

The decision regarding fixation or excision primarily depends on the size or comminuted state of the bony fragment and reducibility of the fracture fragment. A piece of fracture fragment >2 cm can be considered for hip arthroscopy–assisted internal fixation, whereas the others can be removed. The excision group had better outcomes than the fixation group. Hip arthroscopy–assisted internal fixation or excision of bony fragments led to satisfactory short-term clinical and radiological results for the treatment of Pipkin Type I and II femoral head fractures. We observed no major complications after hip arthroscopy.

## Data Availability

The data have been entirely included in the manuscript.

## References

[1] Pipkin G . Treatment of grade IV fracture-dislocation of the hip. *J Bone Joint Surg Am* 1957; 39-A: 1027–42 passim. 13475403

[2] Giannoudis PV, Kontakis G, Christoforakis Z et al. Management, complications and clinical results of femoral head fractures. *Injury* 2009; 40: 1245–51. 19897188 10.1016/j.injury.2009.10.024

[3] Droll KP, Broekhuyse H, O’Brien P. Fracture of the femoral head. *J Am Acad Orthop Surg* 2007; 15: 716–27. 18063712 10.5435/00124635-200712000-00005

[4] Scolaro JA, Marecek G, Firoozabadi R et al. Management and radiographic outcomes of femoral head fractures. *J Orthop Traumatol* 2017; 18: 235–41. 28188487 10.1007/s10195-017-0445-z PMC5585088

[5] Butler JE . Pipkin type-II fractures of the femoral head. *J Bone Joint Surg Am* 1981; 63: 1292–6. 7287800

[6] Park KS, Lee K-B, Na B-R et al. Clinical and radiographic outcomes of femoral head fractures: excision vs. fixation of fragment in Pipkin type I: what is the optimal choice for femoral head fracture? *J Orthop Sci* 2015; 20: 702–7. 25952457 10.1007/s00776-015-0732-6

[7] Mostafa MF, El-Adl W, El-Sayed MA. Operative treatment of displaced Pipkin type I and II femoral head fractures. *Arch Orthop Trauma Surg* 2014; 134: 637–44. 24570143 10.1007/s00402-014-1960-5

[8] Tang Y, Liu Y, Zhu Y et al. Surgical hip dislocation approach for treatment of femoral head fracture. *Zhongguo Xiu Fu Chong Jian Wai Ke Za Zhi* 2015; 29: 1327–31. 26875261

[9] Wang CG, Li Y-M, Zhang H-F et al. Anterior approach versus posterior approach for Pipkin I and II femoral head fractures: a systemic review and meta-analysis. *Int J Surg* 2016; 27: 176–81. 26854958 10.1016/j.ijsu.2016.02.003

[10] Wang S, Li B, Li J et al. Comparison of the modified Heuter approach and the Kocher-Langenbeck approach in the treatment of Pipkin type I and type II femoral head fractures. *Int Orthop* 2019; 43: 2613–20. 30683993 10.1007/s00264-019-04301-5

[11] Guo JJ, Tang N, Yang HL et al. Impact of surgical approach on postoperative heterotopic ossification and avascular necrosis in femoral head fractures: a systematic review. *Int Orthop* 2010; 34: 319–22. 19680651 10.1007/s00264-009-0849-3 PMC2899295

[12] Ganz R, Gill TJ, Gautier E et al. Surgical dislocation of the adult hip: a technique with full access to the femoral head and acetabulum without the risk of avascular necrosis. *J Bone Joint Surg Br* 2001; 83: 1119–24. 11764423 10.1302/0301-620x.83b8.11964

[13] Hsu SL, Chen C-Y, Ko J-Y et al. Hip arthroscopy-assisted reduction and fixation for femoral head fracture dislocations: clinical and radiographic short-term results of seven cases. *J Orthop Surg (Hong Kong)* 2019; 27: 2309499019881865. 10.1177/2309499019881865 31640467

[14] Yamamoto Y, Ide T, Ono T et al. Usefulness of arthroscopic surgery in hip trauma cases. *Arthroscopy* 2003; 19: 269–73. 12627151 10.1053/jars.2003.50033

[15] Hari Krishnan B, Joshi GR, Pushkar A. Arthroscopic removal of intraarticular fracture fragment after fracture dislocation of hip. *Med J Armed Forces India* 2015; 71: S208–10. 26265834 10.1016/j.mjafi.2014.02.010 PMC4529577

[16] Lansford T, Munns SW. Arthroscopic treatment of Pipkin type I femoral head fractures: a report of 2 cases. *J Orthop Trauma* 2012; 26: e94–6. 22534685 10.1097/BOT.0b013e3182323f4f

[17] Thompson VP, Epstein HC. Traumatic dislocation of the hip; a survey of two hundred and four cases covering a period of twenty-one years. *J Bone Joint Surg Am* 1951; 33-A: 746–78. 14850515

[18] Ficat RP . Idiopathic bone necrosis of the femoral head. Early diagnosis and treatment. *J Bone Joint Surg Br* 1985; 67: 3–9. 3155745 10.1302/0301-620X.67B1.3155745

[19] Brooker AF, Bowerman JW, Robinson RA et al. Ectopic ossification following total hip replacement. Incidence and a method of classification. *J Bone Joint Surg Am* 1973; 55: 1629–32. 4217797

[20] Tonetti J, Ruatti S, Lafontan V et al. Is femoral head fracture-dislocation management improvable: a retrospective study in 110 cases. *Orthop Traumatol Surg Res* 2010; 96: 623–31. 20729157 10.1016/j.otsr.2010.03.020

[21] Chen Z, Lin B, Ding Z et al. Treatment of Pipkin type I fracture of femoral head associated with posterior dislocation of the hip. *Zhongguo Xiu Fu Chong Jian Wai Ke Za Zhi* 2011; 25: 521–5. 21675104

[22] Epstein HC, Wiss DA, Cozen L. Posterior fracture dislocation of the hip with fractures of the femoral head. *Clin Orthop Relat Res* 1985; 201: 9–17. 4064426

[23] Brumback RJ, Kenzora JE, Levitt LE et al. Fractures of the femoral head. *Hip* 1987: 181–206. 3546215

[24] Yoon TR, Rowe SM, Chung JY et al. Clinical and radiographic outcome of femoral head fractures: 30 patients followed for 3-10 years. *Acta Orthop Scand* 2001; 72: 348–53. 11580122 10.1080/000164701753541998

[25] Marsh JL, Slongo TF, Agel J et al. Fracture and dislocation classification compendium - 2007: Orthopaedic Trauma Association Classification, Database and Outcomes Committee. *J Orthop Trauma* 2007; 21: S1–133. 18277234 10.1097/00005131-200711101-00001

[26] Chiron P, Lafontan V, Reina N. Fracture-dislocations of the femoral head. *Orthop Traumatol Surg Res* 2013; 99: S53–66. 23357042 10.1016/j.otsr.2012.11.007

[27] Chakraborti S, Miller IM. Dislocation of the hip associated with fracture of the femoral head. *Injury* 1975; 7: 134–42. 1205607 10.1016/0020-1383(75)90011-x

[28] Henle P, Kloen P, Siebenrock KA. Femoral head injuries: Which treatment strategy can be recommended? *Injury* 2007; 38: 478–88. 17400227 10.1016/j.injury.2007.01.023

[29] Epstein HC . Posterior fracture-dislocations of the hip; long-term follow-up. *J Bone Joint Surg Am* 1974; 56: 1103–27. 4436348

[30] Chen ZW, Zhai W-L, Ding Z-Q et al. Operative versus nonoperative management of Pipkin type-II fractures associated with posterior hip dislocation. *Orthopedics* 2011; 34: 350. 10.3928/01477447-20110317-09 21598886

[31] Chen ZW, Lin B, Zhai W-L et al. Conservative versus surgical management of Pipkin type I fractures associated with posterior dislocation of the hip: a randomised controlled trial. *Int Orthop* 2011; 35: 1077–81. 20680276 10.1007/s00264-010-1087-4 PMC3167412

[32] Dowd GS, Johnson R. Successful conservative treatment of a fracture-dislocation of the femoral head. A case report. *J Bone Joint Surg Am* 1979; 61: 1244–6. 511887

[33] Kelly RP, Yarbrough SH 3rd. Posterior fracture-dislocation of the femoral head with retained medial head fragment. *J Trauma* 1971; 11: 97–108. 5099828

[34] Roeder LF Jr., DeLee JC. Femoral head fractures associated with posterior hip dislocation. *Clin Orthop Relat Res* 1980; 147: 121–30. 7371277

[35] Tsai SHL, Tai W-C, Fu T-S et al. Does surgical repair benefit Pipkin type I femoral head fractures?: a systematic review and meta-analysis. *Life* 2022; 12: 71. 10.3390/life12010071 PMC8780341 35054465

[36] Holmes WJ, Solberg. B, Bay BK et al. Biomechanical consequences of excision of displaced Pipkin femoral head fractures. *J Orthop Trauma* 2000; 12: 149–50.

